# Arabincoside B isolated from *Caralluma arabica* as a potential anti-pneumonitis in LPS mice model

**DOI:** 10.1007/s10787-023-01159-3

**Published:** 2023-02-23

**Authors:** Riham A. El-Shiekh, Ghazal Nabil, Aya A. Shokry, Yasmine H. Ahmed, Othman S. S. Al-Hawshabi, Essam Abdel-Sattar

**Affiliations:** 1 Department of Pharmacognosy, Faculty of Pharmacy, Cairo University, Cairo, 11562 Egypt; 2 Department of Pharmacology, Faculty of Veterinary Medicine, Cairo University, Giza, 12211 Egypt; 3 Department of Cytology & Histology, Faculty of Veterinary Medicine, Cairo University, Giza, 12211 Egypt; 4 Department of Biology, Faculty of Science, University of Aden, Aden, Yemen

**Keywords:** Pneumonitis, COVID-19, Arabincoside B, *Caralluma arabica*, Anti-inflammatory

## Abstract

Acute lung injury (ALI) is a life-threatening condition usually associated with poor therapeutic outcomes and a high mortality rate. Since 2019, the situation has worsened due to the COVID-19 pandemic. ALI had approximately 40% of deaths before COVID-19, mainly due to the dysfunction of the blood–gas barrier that led to lung edema, failure of gas exchange, and dyspnea. Many strategies have been taken to mitigate the disease condition, such as diuretics, surfactants, antioxidants, glucocorticoids, heparin, and ventilators with concomitant sedatives. However, until now, there is no available effective therapy for ALI. Thus, we are presenting a new compound termed Arabincoside B (AR-B), recently isolated from *Caralluma arabica,* to be tested in such conditions. For that, the lipopolysaccharide (LPS) mice model was used to investigate the capability of the AR-B compound to control the ALI compared to standard dexamethasone. The results showed that AR-B had a significant effect on retrieving ALI. A further mechanistic study carried out in the serum, lung homogenate, histological, and immunohistochemistry sections revealed that the AR-B either in 50 mg/kg or 75 mg/kg dose inhibited pro-inflammatory cytokines such as IL-6, IL-13, NF-κB, TNFα, and NO and stimulated regulatory cytokines IL-10. Moreover, AR-B showed a considerable potential to protect the pulmonary tissue against oxidative stress by decreasing MDA and increasing catalase and Nrf2. Also, the AR-B exhibited an anti-apoptotic effect on the lung epithelium, confirmed by reducing COX and BAX expression and upregulating Bcl-2 expression. These results pave its clinical application for ALI.

**Supplementary Information:**

The online version contains supplementary material available at 10.1007/s10787-023-01159-3.

## Introduction

Acute pneumonitis/acute lung injury (ALI)/acute respiratory distress syndrome (ARDS) are used interchangeably to describe a life-threatening disease of the lung characterized by acute onset, bilateral infiltration, protein-rich pulmonary edema, hypoxia, hypercapnia associated with worse clinical outcomes (Ragaller and Richter 2010). This condition is mainly associated with characteristic pathological features, including injury of alveolar epithelium and vascular endothelium that eventually leads to alveolar barrier dysfunction (Johnson and Matthay 2010). The ALI either results from non-infectious causes such as trauma, burns, smoking and mechanical ventilation, or infectious causes (Ragaller and Richter 2010) (technically known as pneumonia according to WHO) such as bacteria, fungi, and viruses such as coronavirus. The disease incidence is hard to estimate due to the lack of a decisive definition, clear inclusion/exclusion criteria, and geographical variation (Johnson and Matthay 2010). However, according to Rubenfeld et al. study in 2005, the morbidity was around 200,000 patients/year with a 40% mortality rate in the USA (Johnson and Matthay 2010; Rubenfeld et al. 2005). Anyhow, the life quality of ALI survivors is pathetically affected (Johnson and Matthay 2010).

Since 2019, the master scene has been tragically aggravating due to the coronavirus attack in Wuhan, a city in China. It was defined by WHO as COVID-19 in February 2020 (WHO 2020). In effect, the virus rapidly spread worldwide, causing a pandemic crisis associated with mounting morbidity and mortality rate due to its associated pneumonia (Wiersinga et al. 2020) or ventilator-associated pneumonia occurring among COVID-19 patients (Vacheron et al. 2022). The persistence of respiratory complications post-COVID-19, including pulmonary fibrosis, is a worrisome sequela that could lead to life-long disability or death (Bazdyrev et al. 2021; Zhou et al. 2022). The post-COVID fibrosis associated with fatigue, cough, dyspnea, and exercise intolerance was observed in patients with mild to severe symptoms (Bazdyrev et al. 2021). Despite the tremendous global medical efforts, there are no effective treatment and/or rehabilitation agents to alleviate COVID-associated pulmonary damage (Bazdyrev et al. 2021).

Corticosteroids were one of the available choices against post-COVID-19 pulmonary fibrosis. Prednisone was found to decrease the required home oxygen, limit the interstitial filtrate confirmed by chest X-ray, and improved pulmonary function test (Lam et al. 2021). In contrast, a meta-analysis systemic review by Nader et al. indicated that corticosteroids were ineffective in patients suffering from COVID-19 pneumonia (Ebrahimi Chaharom et al. 2022). In addition, Freitas et al. found that prolonged administration of corticosteroids in COVID-19 was positively correlated with the longer duration of mechanical ventilation and ICU-acquired myopathy (Maia et al. 2022).

However, many clinical trials are needed to address the utility and risks of using corticosteroids to alleviate post-COVID-19 pulmonary fibrosis. Also, it is generally accepted that prolonged administration of corticosteroids is associated with severe side effects such as delaying virus clearance (Maia et al. 2022), steroidal diabetes, cataract, osteoporosis, adrenal insufficiency, growth retardation, hypertension, hyperlipidemia, gastritis, pancreatitis, liver steatosis, retarded wound healing, collagen breakdown, atrophy of lymphoid organs, and increase of infections incidences (Yasir et al. 2022).

Finding a natural alternative that could be used to decrease the severity of ALI and its complications without the harmful side effects of corticosteroids is a critical unmet clinical need. In the same vein, the current hypothesis aimed to evaluate the ability of Arabincoside B (AR-B) isolated from *Caralluma arabica* (*C. arabica*) to suppress the ALI induced by lipopolysaccharide (LPS) in mice model compared to dexamethasone. The *Caralluma* genus belongs to the Asclepiadoideae subfamily under the Apocynaceae family, which is widely spread in dry regions such as Asia, Africa, Europe, etc*.* (Malladi et al. 2018) with many taxonomists have been trying to clarify the ambiguity of *Caralluma* Indian taxonomy. Traditionally, *Caralluma* species were widely used for treating cancer, diabetes, rheumatism, and malarial and trypanosomal infections (Dutt et al. 2012; Qayyum et al. 2018). Arabincoside B is one of the pregnane glycosides extracted in 2022 from *C. arabica* aerial parts by Abdel-Sattar et al*.* (2022).

In the current study, the ability of AR-B to withstand oxidative stress, inhibit the inflammatory mediators, potentiate the inflammatory inhibitors, and retard apoptosis was evaluated in serum and lung. The obtained results were statistically compared to negative and positive controls in addition to the dexamethasone group (long-acting glucocorticoids) to assist the possible application of AR-B against COVID-19 pneumonia or ALI. To the best of our knowledge, this is the first time AR-B has been tested against lung inflammation.

## Materials and methods

### Plant material

*Caralluma arabica* N. E. Br. [Syn. *Desmidorchis arabica* (N. E. Br.) Meve & Liede; *Crenulluma arabica* (N. E. Br.) Plowes] aerial parts were collected in 7-2020 from the Aqan region, Al-Musaimir District, Lahej Governorate, Southern Yemen (13 220 87100 N, 045 830 34400 E). It was authenticated by Dr. Othman S. S. Al-Hawshabi, Associate Professor of Plant Taxonomy and Flora, Department of Biology, Faculty of Science, Aden University, Yemen. The plant was sliced into small pieces and air dried. A voucher specimen (No. 5659) was placed in the Department of Biology, Faculty of Science, University of Aden.

#### Isolation

The aerial parts of *C. arabica* (300 g powder) were extracted with 95% ethanol following the method reported by Abdel-Sattar et al*.* (2022). Part of the remaining water fraction (8 g) was subjected to chromatographic separation over flash silica gel column 60 (DCM-MeOH-H2O, 10:2:0.1) followed by separation of compound AR-B (450 mg) from fraction Fr-13 (1.03 g) by precipitation.

#### Characterization

**Fig. 1:**
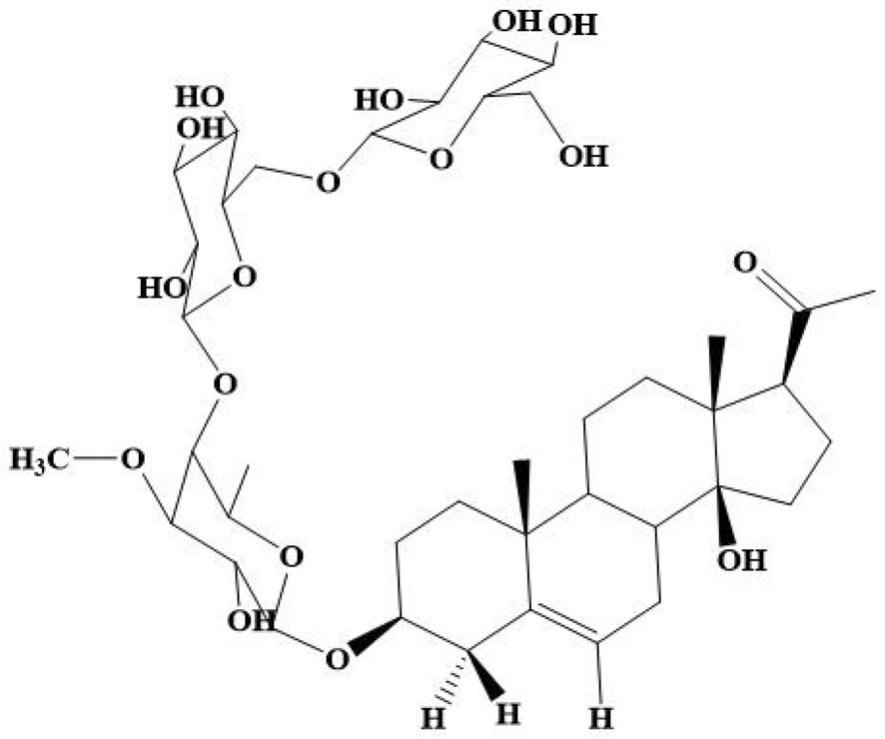
Chemical structure of Arabincoside B

The structure of AR-B was elucidated using spectral means (IR, 1D and 2D-NMR, and ESI–MS) as described by Abdel-Sattar et al*.* (2022) as illustrated in Table 1 and Fig. 1. For more details, check Supplementary Materials.

**Table 1:** The spectral identity

Chemical formula	C_40_H_64_O_17_
Exact mass	816.41
Molecular weight	816.94
*m/z* ratio	816.41 (100.0%), 817.42 (43.3%), 818.42 (9.1%), 818.42 (3.5%), 819.42 (1.5%)

### Induction of in vivo acute pneumonitis

#### Animals

Thirty-five male mice weighing 15–25 g were obtained from the National Research Center, Egypt. Mice were kept under hygienic conditions with a 12-h light/12-h dark cycle, 45–55% relative humidity, and 23–25 °C RT with feed and water available ad libitum. The experiments were carried out according to the Institutional Animal Care and Use Committees (IACUC), Faculty of Veterinary Medicine, Cairo University, Approval No.: Vet CU 2009 2022522. Mice were classified into five groups: seven mice/group given the regime shown in Table 2. The experiment was carried out according to Shokry et al*.* (2022).

**Table 2:** The experimental design

Groups *n* = 7	Negative control (NC)	Positive control (PC)	Standard (DEX)	Arabincoside low dose (ALD)	Arabincoside high dose (AHD)
Oral treatments that were given daily using oral gavage for 1 week	Saline		Dexamethasone 2 mg/kg	Arabincoside 50 mg/kg	Arabincoside 75 mg/kg
			All are dissolved in saline		
Challenge on the seventh day	After 30 min of oral administration of drugs on day 7 of the experiment, the challenge was induced as follows:				
	PBS was instilled intranasally	Lipopolysaccharide (LPS) dissolved in PBS was instilled intranasally at 40 µg/mice dose to induce pneumonitis			
	After 6 h, blood samples were collected from orbital plexuses using heparinized tubes. Then the mice were euthanized, and the lungs were dissected for weighing and further biochemical, histopathological, and immunohistochemical analysis				
Parameters	Serum samples were used to measure catalase, malondialdehyde (MDA), and nitric oxide (NO) levels Lung right lobe homogenates were used to determine interleukins IL-6, IL-10, and IL-13, nuclear factor kappa B (NF-κB), and nuclear factor erythroid 2-related factor 2 (Nrf2) Lung left lobes were used for morphological evaluation by Hematoxylin and Eosin (H&E) staining and for detecting the expression levels of tumor necrosis factor α (TNFα), B cell lymphoma 2 (Bcl-2), BCL2-associated X protein (BAX), and cyclooxygenase 2 (COX 2) using immunohistochemistry (IHC)				

#### In vivo mechanistic studies

##### ELISA

The IL-10, IL-13, NF-κB, and Nrf2 were measured using MyBioSource standard manufactured kits (San Diego, CA, USA) and IL-6 using R&D Systems standard manufactured kit (Minneapolis, MN, USA) in lung homogenate according to the manufacturers’ instructions.

##### Fluorometric

NO and MDA were measured in serum using BioVision standard manufactured kits (CA, USA) according to the manufacturers’ instructions.

##### Spectrophotometric

CAT was measured in serum using MyBioSource standard manufactured kit (San Diego, CA, USA) according to the manufacturers’ instructions.

##### Histological observation by light microscope

The left lobes of the mice’s lungs were quickly removed, inflated, and then fixed with 10% neutral buffered formalin. The fixed samples were dehydrated, followed by xylene, and embedded in paraffin. Sections 3 μm thick were prepared, deparaffinized, and stained with H&E for histopathological examination (Bancroft and Gamble 2008) and IHC. Evaluations of lung injury and inflammatory cell infiltration were conducted using the modified scoring system according to Tianzhu et al*.* (2014).

##### Immunohistochemical examination

- Measurement of inflammatory markers: The TNFα was performed according to Wang et al. (2016) and COX2 according to Ghasemi et al. 2019.
- Measurements of apoptotic markers: The Bcl-2 and BAX were performed according to Rashad et al. (2022).
- Immunohistochemical evaluation by image analysis: Sections stained with anti-TNFα, anti-COX2, anti- Bcl-2, and anti-BAX were analyzed using a digital Leica Quin 500Â image analysis system (Leica Microsystems, Switzerland) housed at the Faculty of Dentistry, Cairo University. The image analyzer was automatically calibrated to convert pixels into units of area (μm2). TNFα, COX-2, Bcl-2, and BAX immunostaining was presented as a percentage of the total area in a standard measuring frame over ten independent fields from different slides in each group at 400× magnification. All areas with positive immunohistochemical staining were evaluated, regardless of the intensity. The mean values and standard error of the mean (SEM) obtained for each specimen were statistically analyzed.

## Results

The isolated compound was white amorphous powder and identified as 3β,14β-dihydroxypregn-5-en-20-one-3-O-β-D-glucopyranosyl-(1/6)-O-β-D-glucopyranosyl-(1/4)-O-β-D-digitalopyranoside; named as Arabincoside B (AR-B).

**Fig. 2:**
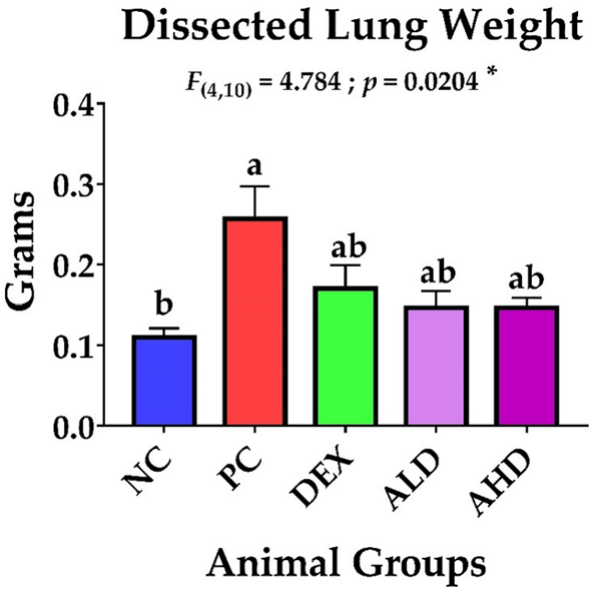
Dissected lung weight. The lungs were dissected from each group after euthanasia and then weighed directly in a sensitive balance. The data were statistically analyzed by one-way ANOVA, followed by the Tukey post hoc test, using GraphPad (Prism). Data were presented as mean ± SEM. Means with different lowercase letters are significantly different among treatments within the same figure

**Fig. 3:**
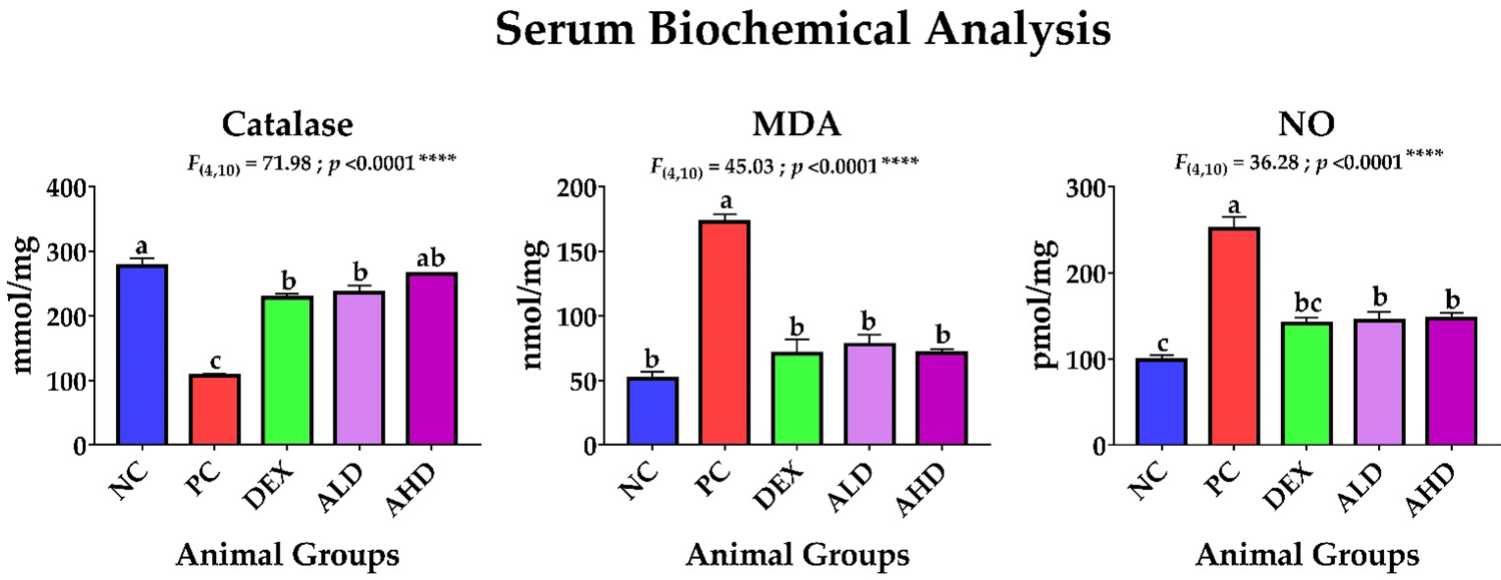
Serum biochemical analysis. Blood was collected from mice orbital plexuses into serum separating tube (SST) for determining the levels of catalase, malonaldehyde (MDA), and nitric oxide (NO). The data were statistically analyzed by one-way ANOVA, followed by the Tukey post hoc test, using GraphPad (Prism). Data were presented as mean ± SEM. Means with different lowercase letters are significantly different among treatments within the same figure

**Fig. 4:**
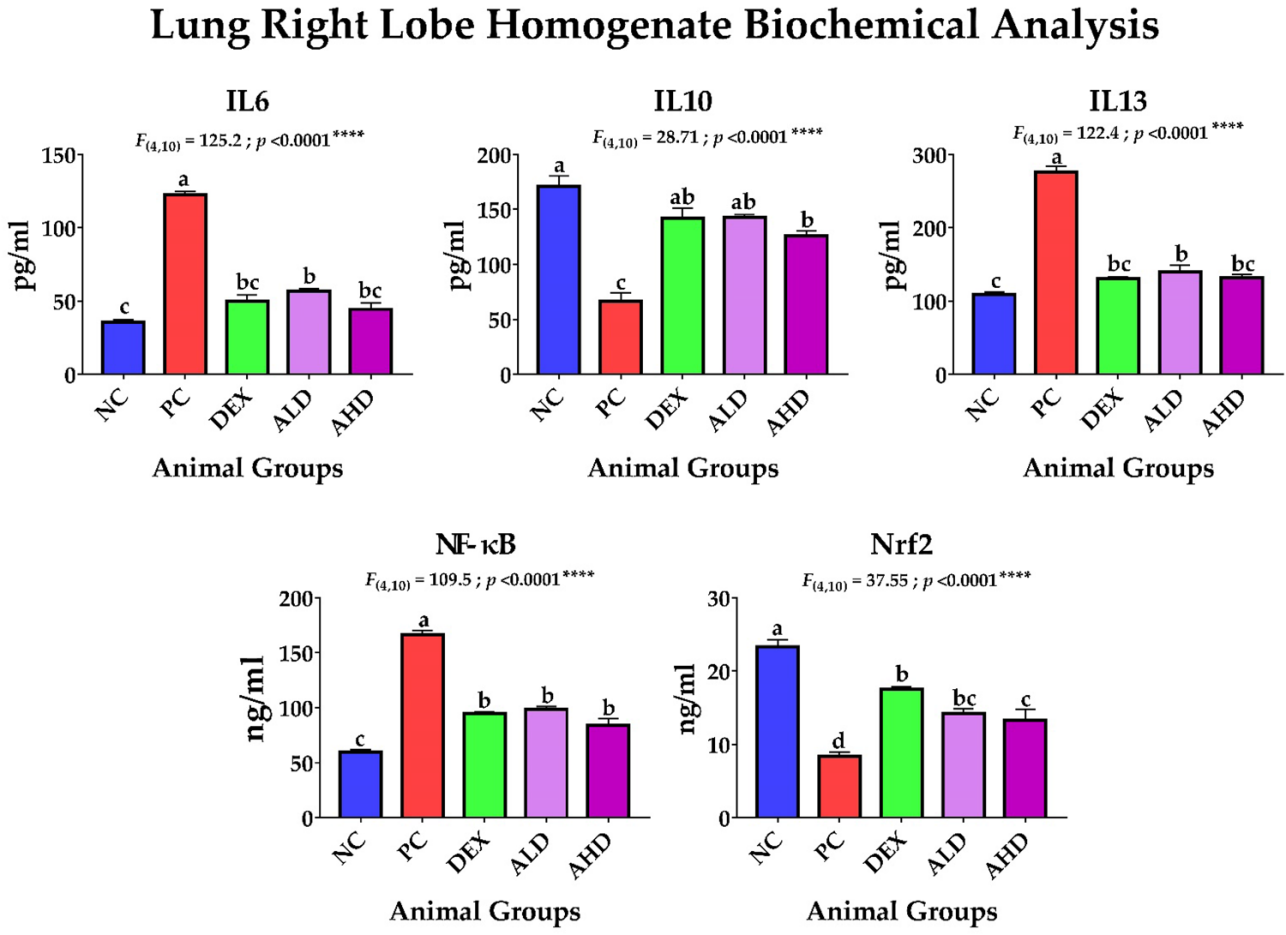
Lung right lobe homogenate biochemical analysis. The lung right lobe was collected from mice in different groups, homogenized for measuring the levels of interleukins (IL) 6, 10, and 13, nuclear factor kappa B (NF-κB), and nuclear factor erythroid 2-related factor 2 (Nrf2). The data were statistically analyzed by one-way ANOVA, followed by the Tukey post hoc test, using GraphPad (Prism). Data were presented as mean ± SEM. Means with different lowercase letters are significantly different among treatments within the same figure

Our study revealed that AR-B extracted from *C. arabica* had a marked effect in controlling the inflammatory process and oxidative stress in mice with ALI with no significant difference between low (ALD) or high dose (AHD). Furthermore, the dissected lungs of euthanized mice in all groups were weighed, where there was a noticeable shrinkage in the lung weight in the dexamethasone (DEX) and both ALD and AHD compared to the PC group, as shown in Fig. 2. Evidently, both doses were able to inhibit the inflammatory process by suppressing the inflammatory promotors, including NO, IL-6, IL-13, and NF-κB, and enhancing the secretion of inflammatory restrictor IL-10, as shown in Figs. 3 and 4. Moreover, both doses were able to tip the balance of antioxidants, including catalase and Nrf2, over oxidative stressor MDA, as shown in Figs. 3 and 4. These results were significantly better than the PC group and paralleled with NC and DEX groups confirming that AR-B had the same potency as dexamethasone either in low or high doses in both blood and site of inflammation, lung.

**Fig. 5:**
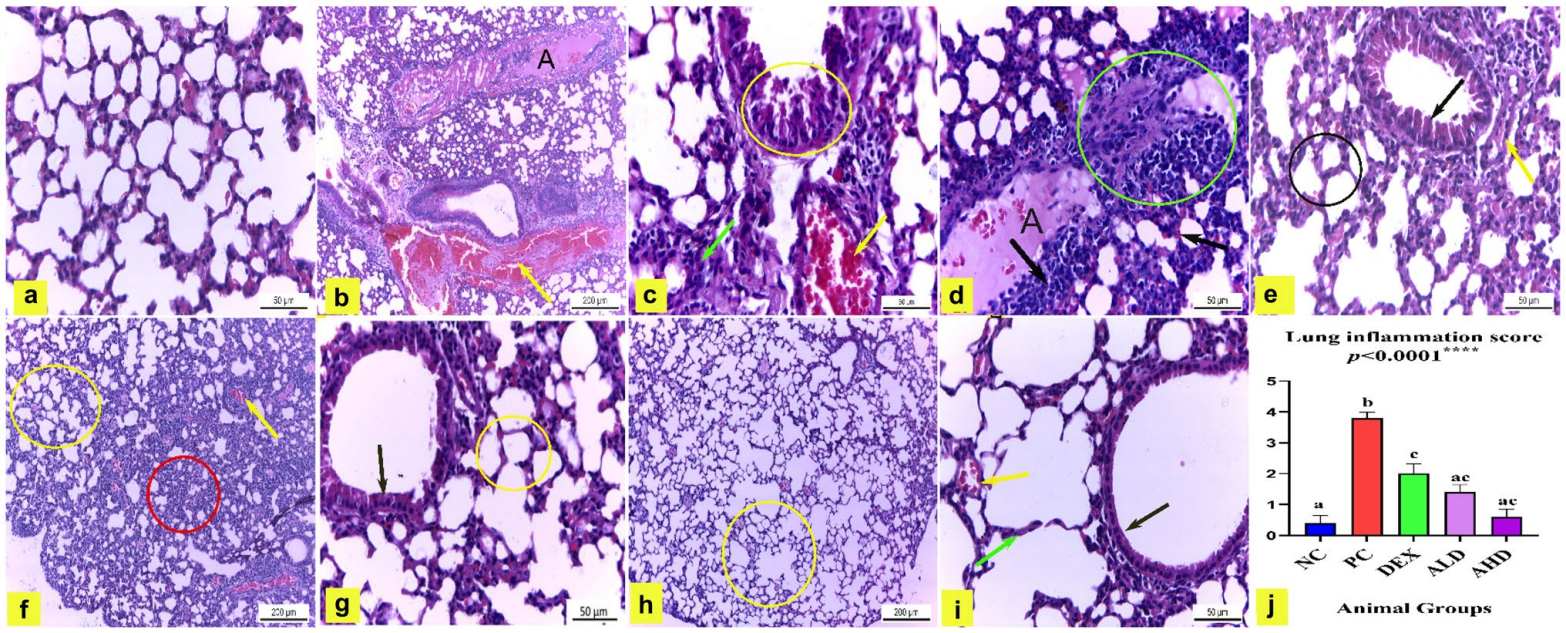
Lung tissue of mice stained with H&E. **a** Negative control mice (NC) showing normal pulmonary histological structure. H&E X400. **b**: **d** Positive control mice (PC) treated with LPS revealing; **b** amyloidosis (A), hemorrhage (yellow arrow). H&E X100. **c** Thick alveolar wall (green arrow); dilated and congested pulmonary capillary (yellow arrow) and degenerated bronchiolar lining epithelium (circle). H&E X400. **d** Infiltration of inflammatory cells (circle).H&E X400. **e** Lung tissue of mice treated with LPS plus dexamethasone (DEX) exhibiting decreased inflammatory cells infiltration (circle) restored the normal lining epithelium of bronchioles (black arrow), narrower and less congested pulmonary vessels (yellow arrow). H&E X400. **f**: **g** Pulmonary tissue of mice treated with LPS plus AR-B low dose (50 mg/kg) (ALD) showing; **f** reduced inflammatory area (circle), narrow and less congested blood vessels (yellow arrow).H&E X100. **g** Normal bronchiolar lining epithelium (arrow) and nearly normal alveolar wall thickness (circle). H&E X400. **h**: **i** Lung tissue of mice treated with LPS plus AR-B high dose (75 mg/kg) (AHD) revealing; **h** pulmonary injury recovery with rare to no inflammatory area (circle). H&E X100. **i** Normal alveolar wall thickness (green arrow) and normal lining epithelium of bronchiole (black arrow). H&E X400. **j** Lung inflammation score. The results were expressed as mean ± SEM. Means with different lowercase letters are significantly different among treatments within the same figure. *P* value ˂0.0001

The observations of H&E-stained lung tissue of the NC showed normal pulmonary histological structure, as shown in Fig. 5a. However, lung tissue obtained from the PC treated with LPS revealed various histopathological changes, including amyloidosis and hemorrhage, as shown in Fig. 5b. Moreover, the PC group had degenerated bronchiolar epithelium, thick alveolar wall, and dilated pulmonary vessels that were congested with blood, as shown in Fig. 5c, in addition to infiltration of inflammatory cells as shown in Fig. 5d. Contrariwise, lung tissue of mice treated with LPS plus DEX exhibited an obvious reduction of histopathological alterations evidenced by a decrease in the inflammatory cells infiltration, restoring the normal lining epithelium of bronchioles, pulmonary vessels became narrower and less congested as shown in Fig. 5e. On the other hand, the pulmonary tissue of mice treated with LPS plus ALD showed ameliorated lung injury in the form of reduced inflammatory area, narrow, less congested blood vessels as shown in Fig. 5f, nearly normal bronchiolar lining epithelium and normal alveolar wall thickness were observed as shown in Fig. 5g. Meanwhile, lung tissue of mice treated with LPS plus AHD revealed pulmonary injury recovery evidenced by rare to no inflammatory area as shown in Fig. 5h, normal alveolar wall thickness and normal lining epithelium of bronchiole as shown in Fig. 5i. In addition, the mean histopathological score was significantly increased in the PC compared to the NC group (*P* < 0.0001). However, the histopathological score was significantly decreased by treatment of DEX, and Arabincoside either in low or high doses compared to the PC group (*P* < 0.0001), as presented in Fig. 5j. Overall, the Arabincoside, either in low or high doses, can maintain the pulmonary architecture, relieve congestion, and mitigate inflammation with potency equal to standard dexamethasone.

**Figs. 6, 7:**
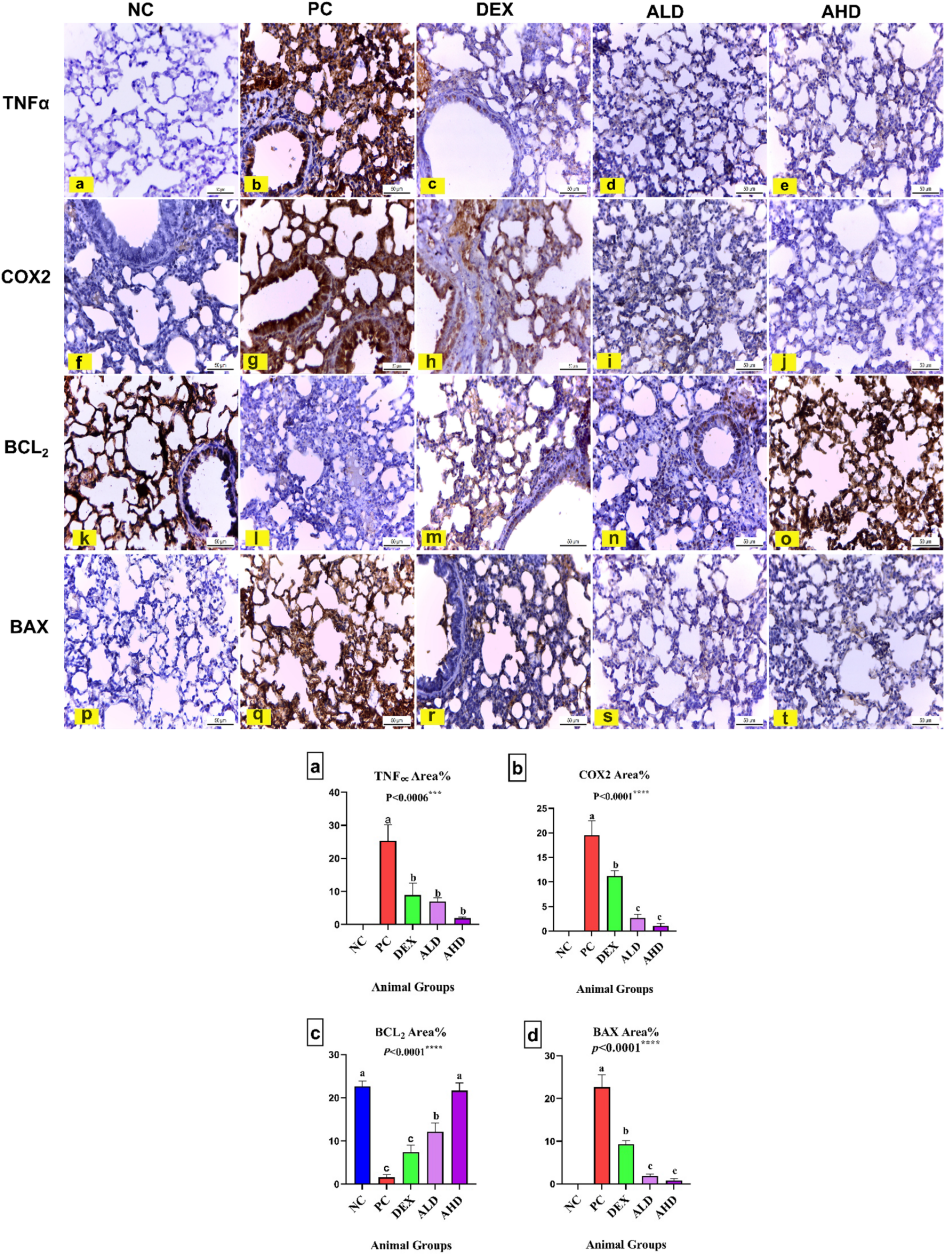
Immunohistochemical analysis of TNFα, COX2, Bcl-2, and BAX. The TNFα (**a**: **e**) and COX2 (**f**: **j**) stained pulmonary tissue (X400). **a** and **f** NC mice had negligible TNFα and COX2 reactions. **b** and **g** PC mice had strong positive TNFα and COX2 immuno-expression. **c** and **h** Lung tissue of mice treated with LPS plus DEX showed a significant decrease in TNFα and COX2 immuno-expression compared to PC. **d** and **i** Mice treated with LPS plus ALD revealed a substantial reduction in TNFα and COX2 immune reactivity in the lung compared to PC. **e** and **j** Mice treated with LPS plus AHD revealed negligible TNFα and COX2 immune reactivity in the lung. **k**: **o** Immunohistochemically Bcl-2-stained pulmonary tissue (X400). **k** Lung tissue of NC mice was a strong positive. **l** Pulmonary tissue of PC mice showed significantly reduced Bcl-2 immunoreactivity. **m** Lung tissue of mice treated with LPS plus DEX had a non-significant increase in Bcl-2 expression. **n** Bcl-2 immunoreaction was significantly increased in the lung of mice treated with LPS plus ALD. **o** Lung tissue of LPS plus AHD-treated mice revealed significantly strong Bcl-2 reactivity. **p**: **t** Immunohistochemically BAX-stained pulmonary tissue (X400). **p** Lung tissue of NC mice had negligible immuno-expression of BAX. **q** PC mice had significantly strong positive BAX immunoreaction. **r** Mice treated with LPS plus DEX showed significantly reduced BAX expression. **s** and **t** Administration of ALD and AHD to mice treated with LPS significantly decreased to negligible BAX immune expression. The immuno-expression intensities were analyzed by the image analyzer of the Leica microscope, as shown in **a**, **b**, **c**, and **d**, at which the effects of LPS, LPS plus DEX, LPS plus ALD and AHD on the percent area covered by TNFα, COX2, Bcl-2_,_ and BAX positive immunoreactive cells are, respectively, presented, within the lung of mice. Results are presented as mean ± SEM. Means with different lowercase letters are significantly different among treatments within the same figure

The NC showed negative immuno-expression of TNFα and COX2 in pulmonary tissue (Fig. 6a, f and Fig. 7a, b). On the other hand, a significant cytoplasmic expression of TNFα and COX2 was noted in lung tissue of the PC by 25.3 and 19.5, respectively, compared to NC group, as shown in Fig. 6b, g and Fig. 7a, b. However, there was a significant decrease of TNFα and COX2 immunoreaction in the pulmonary tissue of mice treated with LPS plus DEX by 8.900 and 11.17, respectively, compared to the PC group, as shown in Fig. 6c, h and Fig. 7a, b. Moreover, there was a significant reduction of TNFα and COX2 immune reaction in lung tissue of mice treated with LPS plus ALD by 6.9 and 2.6, respectively, compared to the PC group, as shown in Fig. 6d, i and Fig. 7a, b. Finally, pulmonary tissue of LPS plus AHD-treated mice showed significantly negligible TNFα and COX2 immuno-expression by 1.9 and 1, respectively, compared with the PC group, as shown in Fig. 6e, j and Fig. 7a, b.

The anti-apoptotic Bcl-2 immuno-expression in lung tissue of NC mice was strongly positive, as shown in Fig. 6k and Fig. 7c. However, the pulmonary tissue of PC mice showed significantly reduced Bcl-2 immunoreactivity by 1.6 compared to NC mice, shown in Fig. 6l and Fig. 7c. Lung tissue of mice treated with LPS plus DEX had a non-significant increase in Bcl-2 expression by 7.4 compared to PC mice (Fig. 6m and Fig. 7c). Contrariwise, Bcl-2 immunoreaction was significantly increased in the lung of mice treated with LPS plus ALD by 12.17 compared to PC mice (Fig. 6n and Fig. 7c). Finally, lung tissue of LPS plus AHD-treated mice revealed significantly strong Bcl-2 reactivity by 21.6 compared to PC mice (Fig. 6o and Fig. 7c).

NC mice’s lung tissue had negligible BAX immuno-expression, the pro-apoptotic marker (Fig. 6p and Fig. 7d). Meanwhile, the pulmonary tissue of PC mice had significantly strong positive BAX immunoreaction by 22.6 compared to NC mice (Fig. 6q and Fig. 7d). However, mice treated with LPS plus DEX showed significantly reduced BAX expression in lung tissue by 9.3 compared to PC mice (Fig. 6r and Fig. 7d). Conclusively, administering Arabincoside low and high doses to mice treated with LPS resulted in a significant decrease to negligible BAX immune expression in lung tissue by 1.8 and 0.8 respectively, compared to PC mice (Fig. 6s, t and Fig. 7d).

From the illustrated statistically analyzed sections, Arabincoside, either in low or high doses, has the same potency as dexamethasone in inhibiting TNFα, but it outperformed dexamethasone in inhibiting COX2 and BAX as well as in stimulating Bcl-2.

## Discussion

The ALI that was first defined by Ashbaugh et al*.* in 1967, followed by extensive research to reduce the mortality rate and improve recovery outcomes, still lacks the appropriate intervention (Suratt and Parsons 2006). Although the disease has many underlying causes, the main feature is the dysfunction of the blood–gas barrier, which eventually leads to profound proteinous alveolar edema; in turn, the gas exchange is hindered. One of ALI’s hallmarks is the presence of intact and injured neutrophils with its proteolytic and oxidative content, which positively correlates with mortality rates (Suratt and Parsons 2006). The recruitment of neutrophils to the lung is through many cytokines. First, the activated macrophages and monocyte release a wave of pro-inflammatory cytokines, including tumor necrosis factor-α (TNF-α), interleukin-1 β (IL-1β), interleukin-6 (IL-6), and interleukin-8 (IL-8) (Suratt and Parsons 2006). Those are responsible for fever, migration of immune cells to the lung, especially neutrophils, cardinal signs of local inflammation, and release of acute phase proteins (Dinarello 2000; Jain et al. 2011) in addition to widening of intra-endothelial junctions, which enable neutrophils margination to air spaces (Suratt and Parsons 2006). Then these neutrophils release their reactive oxygen species (ROS) and nitric oxide (NO), which is termed oxidative burst or respiratory burst, which contributes to damage to pulmonary epithelium and vascular endothelium (Chen and Junger 2012; Galkina et al. 2019; Chen et al. 2008). Subsequently, the damaged cells produce procoagulant and fibrinolysis molecules which leads to the deposition of fibrin and subsequent formation of microvascular thrombi and fibrin-rich proteinaceous casts in the alveoli called intra-alveolar hyaline membranes, which is characteristic to ALI (Suratt and Parsons 2006). Moreover, the pressure of the flooded proteinous edematous fluid also contributes to alveolar cell type II damage, leading to alveolar collapse due to improper production of surfactant, causing physiological shunting and hypoxia (Suratt and Parsons 2006). Without a correct intervention, the fibroproliferative phase will be the only consequence characterized by the migration of myofibroblasts into the alveoli and subsequent lung fibrosis by the fifth to seventh day (Suratt and Parsons 2006).

Ventilators play a double-edged sword as they can induce ALI by alveolar shearing (Suratt and Parsons 2006); however, during emergencies, they could be the difference between life and death. The attack of COVID-19, of course, has worsened the situation by increasing the number of ALI cases (Gibson et al. 2020) along with limited available ventilators as they are bulky, expensive, and require extensive training and concomitant sedative administration.

From the aforementioned mechanism, the early-stage blockage of cytokine-mediated neutrophil recruitment is the best therapeutic approach in ALI. Thus, we present the AR-B compound, which has shown promising results in the early-stage ALI induced by LPS in mice. The LPS model was selected in this model as in PC; the LPS was able to generate various acute lung injury signs (Jing et al. 2015), including hemorrhage and inflammatory cells infiltration (Shokry et al. 2022; Shen et al. 2020; Tian et al. 2019; Jiang et al. 2017) in addition to degeneration of bronchiolar epithelium and endothelial cells and thickening of the alveolar wall (Chen et al. 2022; Fan et al. 2018) leading to disturbance of alveolar–capillary barrier similar to what happens in ALI.

In this study, we found that the AR-B was parallel to DEX and acts on three axes: (1) terminate the inflammatory response, (2) decrease the lung edema, and (3) maintain or restore the pulmonary tissue architecture. The termination of the inflammatory process was supported by a marked decrease in IL-6, TNF-α, and NF-κB, which are known to enhance inflammation in early ALI (Suratt and Parsons 2006; Devaney et al. 2013) and increase anti-inflammatory cytokines IL-10, which was found to reduce neutrophilic activity and inflammatory mediators in ALI in LPS mice model (Inoue 2000) compared to PC group. Second, the reduction of pulmonary edema was proved by decreasing lung weight of AR-B-treated groups associated with a potent decrease in NO and COX production, which leads to narrowing pulmonary vessels. That mitigates their role in pulmonary vasculature dilation, exudation, pain, and fever, which promotes the application of nitric oxide synthetase inhibitors (Akgül et al. 2019) and glucocorticoids (Marik et al. 2011) as possible strategies for alleviating ALI. Moreover, a significant decrease in IL-13, responsible for mucus hypersecretion, goblet cell metaplasia, non-specific airway hyperreactivity, fibrosis, IgE production, and asthma (Doran et al. 2017), pave the ability of AR-B to be used in IL-13 restraining approach to overcome lung fibrosis (Chung et al. 2016). Finally, maintenance of the lung tissue was confirmed by histopathology in both DEX and AR-B groups. In these groups, there is a marked reduction in histopathological alterations, rare to no inflammatory area, normal alveolar epithelium lining and thickness, and narrower and less congested pulmonary vessels. Our dexamethasone results are consistent with its clinical recommendation for various inflammatory diseases (Tian et al. 2019; Rhen and Cidlowski 2005). The ability of AR-B to protect lung tissue from lipid peroxidation was supported by a distinctive decrease in MDA and increase in catalase andNrf2 levels which have been found to protect the lung epithelium and vascular endothelium against oxidative stress that can induce tissue destruction and irreversible fibrosis (Ward 2010; Mokra and Mokry 2019). Additionally, the AR-B compound was found to inhibit the apoptosis cascade by downregulating BAX (pro-apoptotic) and upregulating Bcl-2 expressions (anti-apoptotic), as demonstrated in IHC sections, preventing lung injury. BAX induces apoptosis by creating pores in the mitochondrial membrane facilitating the release of cytochrome c into the cytosol to initiate the cellular apoptosis cascade (Kolliputi and Waxman 2009).In human patients with diffuse alveolar damage, pneumocyte type I and II undergo apoptosis accompanied by DNA fragmentation, which was mediated by BAX and Fas upregulation, especially in acute severe cases; thus, anti-apoptotic therapeutics would be helpful in early ALI to prevent cellular destruction (Matute-Bello and Martin 2003). However, apoptosis is mandatory in the late stage of ALI to remove cellular debris and hyaline membrane, which is essential to restore lung architecture and gas exchange process (Suratt and Parsons 2006).

Our results support that Arabincoside B (AR-B), pregnane glycosides recently isolated from *C. arabica,* can suppress the ALI induced by LPS in a mice model comparable with standard dexamethasone. Our findings are supported by early work carried out in 2001 and 2002 using a 10% ethanolic extract of *C. arabica*, which showed central and peripheral antinociceptive, anti-inflammatory (Zakaria et al. 2001), cytoprotective and anti-gastric ulcer (Zakaria et al. 2002) effects of this extract.

## Conclusion

Conclusively the isolated Arabincoside B (AR-B) has shown a promising compound to alleviate ALI in its early stage through three main axes, including controlling the pulmonary inflammation, reducing pulmonary edema and maintaining the pulmonary architecture, which would further keep the blood–gas barrier integrity.

## Supplementary Information

Below is the link to the electronic supplementary material.

Supplementary file1 (DOCX 575 KB)

## Data Availability

Enquiries about data availability should be directed to the authors.
