# Editorial Expression of Concern: Arabincoside B isolated from *Caralluma arabica* as a potential anti‑pneumonitis in LPS mice model

**DOI:** 10.1007/s10787-025-02057-6

**Published:** 2025-12-05

**Authors:** Riham A. El‑Shiekh, Ghazal Nabil, Aya A. Shokry, Yasmine H. Ahmed, Othman S. S. Al‑Hawshabi, Essam Abdel‑Sattar

**Affiliations:** 1 Faculty of Pharmacy, Department of Pharmacognosy, Cairo University, Cairo, 11562 Egypt; 2 Faculty of Veterinary Medicine, Department of Pharmacology, Cairo University, Giza, 12211 Egypt; 3 Faculty of Veterinary Medicine, Department of Cytology & Histology, Cairo University, Giza, 12211 Egypt; 4 Faculty of Science, Department of Biology, University of Aden, Aden, Yemen

**Editorial Expression of Concern to:Inflammopharmacology (2023) 31:1437–1447** 10.1007/s10787-023-01159-3

The Editors-in-Chief would like to alert readers that there are some concerns related to this article. Concern has been raised regarding figures 6e and 6s being duplicate images and figures 6i and 6t being overlapping images. Images in this paper also appear to overlap with images in another article [1], with figure  6a in this article overlapping with figure 7a [1] and figure 6g in this article with figure 7b [1]. The authors provided the corrected images, however the journal was unable to validate these. Readers are advised to interpret the details of this article with caution.

Riham A. El-Shiekh, Yasmine H. Ahmed, Othman S. S. Al-Hawshabi and Essam Abdel-Sattar all agreed with this editorial expression of concern. Ghazal Nabil and Aya A. Shokry did not disagree with this editorial expression of concern but did not fully agree with the wording.

1. Eloutify, Y.T., El-Shiekh, R.A., Ibrahim, K.M. et al. Bioactive fraction from Plumeria obtusa L. attenuates LPS-induced acute lung injury in mice and inflammation in RAW 264.7 macrophages: LC/QToF-MS and molecular docking. Inflammopharmacol 31, 859–875 (2023).

