# Supplementary material for: Arabincoside B isolated from Caralluma arabica as a potential anti-pneumonitis in LPS mice model
Source: Inflammopharmacology. 2023 Feb 23;31(3):1437–47. doi: 10.1007/s10787-023-01159-3 (PMC9948789; doi:10.1007/s10787-023-01159-3)
Supplement: Supplementary file 1 — Supplementary file1 (DOCX 575 KB) [file 10787_2023_1159_MOESM1_ESM.docx]

**Supplementary materials**

**Arabincoside B Isolated from** ***Caralluma arabica* as a Potential Anti** **Pneumonitis in LPS Mice Model**

**Riham A. El-Shiekh^1#*^, Ghazal Nabil^2#*^, Aya A. Shokry^2#^, Yasmine H. Ahmed^3#^, Othman S. S. Al-Hawshabi^4^, Essam Abdel-sattar^1^**

**^1^** Department of Pharmacognosy, Faculty of Pharmacy, Cairo University, Cairo, Egypt,11562.

**^2^** Department of Pharmacology, Faculty of Veterinary Medicine, Cairo University, Giza, Egypt,12211.

**^3^** Department of Cytology and Histology, Faculty of Veterinary Medicine, Cairo University, Giza, Egypt,12211.

**^4^** Department of Biology, Faculty of Science, University of Aden, Aden, Yemen.

# Authors are of equal contributions

*** Correspondence is addressed to:** [**riham.adel@pharma.cu.edu.eg**](mailto:riham.adel@pharma.cu.edu.eg) **; Tel.:** **+201064763764 &** [**Gazal_nabil@cu.edu.eg**](mailto:Gazal_nabil@cu.edu.eg) **; Tel.: +201020324536.**

1. **Experimental section**
   1. *General experimental procedures*

 Optical rotations were measured on a Bellingham + Stanley ADP 440 + digital polarimeter (Bellingham & Stanley, Kent, UK). NMR was conducted on Bruker High Performance Avance III FT-NMR spectrometer (^1^H-NMR: 400 MHz and ^13^C-NMR: 100 MHz) and using TMS as internal standard. IR spectrophotometer, Shimadzu FT-IR Affinity-1 was used for recording IR spectra using KBr discs.  The electron spray ionization-mass spectroscopy (ESI-MS) was performed on PlateExpress TLC plate reader coupled to the Expression compact mass spectrometer (CMS) (Advion, Ithaca, NY, USA). The Advion is a single-quadrupole mass spectrometer that provides electrospray ionization (ESI) in both in the positive and negative ionization. Analytical TLC was carried out on Merck TLC plates KGF Silica gel 60 and KGF RP-18 Silica gel 60 and spots were visualized under UV light (254 and 365 nm) and after spraying with *p*-anisaldehyde/H_2_SO_4_ followed by heating at 110 °C. Column chromatography (CC) was carried on flash silica gel 60 (Merck, particle size 230–400 mesh), and RP-C18 (silica gel, 40-63 µm; Merck).

- 1. *Arabincoside B*

White amorphous powder, [α]^D^_21_ -98.52 (*c*. 0.10, MeOH); IR *υ*_max_ (KBr, cm^‐1^): 3379, 2935, 1681, 1419, 1361, 1346, 1284, 1176, 1049, 964, 763 and 640; Table S1 for ^1^H and ^13^C NMR arabincoside B (400 MHz, 100 MHz, CH_3_OH-d_6_) of aglycone and sugar moieties; Figures S1 and S2 for ^1^H and ^13^C NMR spectra of arabincoside B; ESI–MS, m/z (rel. int.): 839.1 [M+Na]^+^ (100) in positive mode, and 815.0 [M-H]^-^ in negative mode.

**Table S1. ^1^H- and ^13^C-NMR spectral data of arabincoside B (DMSO-d_6_)**

|  | **^1^H-NMR** | **^13^C-NMR** |  | **^1^H-NMR** | **^13^C-NMR** |
| --- | --- | --- | --- | --- | --- |
| **No.** |  |  | **No.** | **Dig** | |
| 1 | 1.01 (1H, *m*), 1.80 (1H, *m*) | 37.18 | 1` | 4.20 (1H, *d*, *J*= 7.60) | 101.75 |
| 2 | 1.48 (2H, *m*) | 29.76 | 2` | 3.58 (1H, *m*) | 69.11 |
| 3 | 3.40 (1H, *m*) | 77.58 | 3` | 3.07 (1H, *m*) | 84.49 |
| 4 | 1.52 (1H, *m*), 2.35 (1H, *dd*, *J*= 2.84, 10.48) | 38.71 | 4` | 4.05 (1H, *b.s*) | 74.00 |
| 5 | - | 139.66 | 5` | 3.52 (1H, *m*) | 69.75 |
| 6 | 5.35 (1H,  *br d, J*= 5.24) | 122.19 | 6` | 1.13 (3H, *d*, *J*= 6.2) | 17.62 |
| 7 | 1.74 (1H, *m*), 2.16 (1H, *m*) | 27.40 | OCH_3_ | 3.38 (3H, *s*) | 58.22 |
| 8 | 1.58 (1H, *m*) | 37.14 | **Glc** | | |
| 9 | 1.10 (1H, *m*) | 45.89 | 1`` | 4.28 (1H, *d*, *J*= 7.72) | 103.50 |
| 10 | - | 36.98 | 2`` | 2.95 (1H, *m*) | 74.63 |
| 11 | 1.32 (1H, *m*), 1.43 (1H, *m*) | 20.82 | 3`` | 3.05 (1H, *m*) | 77.19 |
| 12 | 1.34 (1H, *m*), 2.12 (1H, *m*) | 38.85 | 4`` | 3.07 (1H, *m*) | 70.92 |
| 13 | - | 49.07 | 5`` | 3.31 (1H, *m*) | 76.82 |
| 14 | - | 84.62 | 6`` | 3.97 (1H, *d*, *J*= 11.64), 3.58 (1H, *m*) | 69.47 |
| 15 | 1.93 (1H, *m*), 1.61 (1H, *m*) | 33.85 | **Glc** | | |
| 16 | 1.83 (1H, *m*), 1.93 (1H, *m*) | 23.79 | 1``` | 4.36 (1H, *d*, *J*= 7.68) | 103.92 |
| 17 | 2.72 (1H, *dd*, *J*= 4.8, 9.2) | 62.99 | 2``` | 2.98 (1H, *m*) | 73.96 |
| 18 | 0.88 (3H, *s*) | 15.70 | 3``` | 3.05 (1H, *m*) | 77.19 |
| 19 | 0.93 (3H, *s*) | 19.66 | 4``` | 3.02 (1H, *m*) | 70.52 |
| 20 | - | 215.61 | 5``` | 3.10 (1H, *m*) | 77.36 |
| 21 | 2.20 (3H, *s*) | 31.94 | 6``` | 3.67 (2H, *dd, J*= 5.6, 11.60) | 61.54 |

Arabincoside B

**Figure S1. ^1^H-NMR spectrum of arabincoside B (DMSO-d_6_)**

Arabincoside B

**Figure S2. ^13^C-NMR spectrum of arabincoside B (DMSO-d_6_)**
